# Some Unusual Operations on Lunatics and Their Results
1Read at the meeting of the Bristol Medico-Chirurgical Society, on April 12th, 1899.


**Published:** 1899-09

**Authors:** J. Paul Bush

**Affiliations:** Surgeon to the Bristol Royal Infirmary.


### SOME UNUSUAL OPERATIONS ON LUNATICS AND THEIR RESULTS.[1]

BY

J. Paul Bush, M.R.C.S. Eng.,

*Surgeon to the Bristol Royal Infirmary.*

---

I have ventured to publish these five operation cases occurring in persons of unsound mind, as the cases are interesting not only to those working at surgery but also to those whose practice lies more especially in the treatment and diagnosis of mental cases; the recording of these cases may also raise the question, "What effects (if any) are produced on the mental condition after major operations on the insane?" These cases have all occurred within the past year, and I am glad to say, from a surgical standpoint, I am quite satisfied with the results. As time slips by one is rather apt to remember cases that have done well, while those that have done badly sometimes escape our memory. I therefore mention that the operations described below were consecutive ones, and in all of them improvement in the mental condition commenced shortly after operation, and this improvement continued till apparently the brain was restored to a healthy state, which, so far, has continued to be permanent in every case. In none of these operation cases was the bodily ailment even supposed to be a starting point for the deranged mental faculties, as in all the patients the unsound condition of the mind was manifest for months or years before the condition of the body, requiring the interference of the surgeon, was present.

E. K., a young lady whose mind had become unhinged,—it was thought through a love affair,—and who had been under restraint for three years, had two bad ingrowing toe-nails removed; she completely recovered her mental condition within three months.

G. D., a man aged 70, with cystitis and dysuria, was found to have a stone in his bladder. I operated by the supra-pubic method, and

[1] Read at the meeting of the Bristol Medico-Chirurgical Society, on April 12th, 1899.

extracted a calculus two inches long and one inch thick; in the centre of the stone was found a vulcanite pipe-stem. I had the bladder drained for ten days, on account of the cystitis; the abdominal wound was completely healed by the 17th day, the mental condition began to improve at once, and he was cured in body and mind within six months.

A. W., aged 44. This woman had been in an asylum for 2½ years with melancholia and severe suicidal impulses. On admission her physical condition was normal, a year after she had retention of urine and intermittent attacks of menorrhagia which continued for six months; when I saw her she was very anæmic, there was a large solid tumour reaching from the pubes to the level of the umbilicus, the os uteri was easily dilated, and I was able to reach and cut through a broad short pedicle attached to the side of the uterus. My trouble now began: the corkscrews used in the extraction of fibroids held well, but I was quite unable, on account of its size, to take away the tumour, which was now free in the uterus: as hemorrhage was taking place, I was afraid I might have, after all, to do an abdominal section and lay open the uterus, but by the timely arrival of a pair of midwifery forceps, and the exercise of some muscular energy, I was enabled at last to deliver the tumour, which, emptied of its blood, was somewhat larger in size than that of the fœtal head at the full time. The uterus was packed with iodoform gauze to stop hemorrhage; she made an uninterrupted recovery and was discharged mentally cured in some six months.

M. D., a woman aged 46. When first she came under notice there was nothing abnormal in her bodily condition; mentally she was acutely maniacal. When I saw her there was some history of a slight attack of jaundice seven weeks before, followed two weeks later by a sudden severe pain at the epigastrium, with temporary collapse, and afterwards a rise of temperature with diarrhœa: for ten days she seemed better, when the temperature suddenly rose to 104°, rigors came on, and a swelling was made out in the epigastrium, which was dull on percussion with an area of resonance above. I made a free incision, and after letting out about a pint of pus, I found the intestines matted together, the abscess sac being situated between the stomach and liver; I endeavoured to find what was evidently the cause of the abscess, namely, a perforated gastric ulcer; but the stomach was so coated by thick layers of lymph, that I contented myself with getting away as much of the septic *débris* as possible, sponged out the cavity, and packed it with iodoform gauze, leaving the ends outside the wound to act as drainage. The patient was much collapsed, and I must say I expected to hear she had died of septic peritonitis within the twenty-four hours. After some three weeks, during which time there were several attacks of sickness, the vomit containing pus and a watery fluid of a beautiful blue colour, she eventually made a complete recovery, and was mentally cured and discharged some seven months later.

This case was without doubt one of gastric perforation that had formed a residual abscess; whether the perforation was of traumatic origin, or whether it was originally an ulcer that had perforated, it is, I think, impossible to say. At my operation I looked for, but could not find, any trace of a foreign body which might have been the cause of the perforation.

M. R., aged 32. A woman suffering from melancholia with severe suicidal and homicidal impulses, had been under treatment for two years. A year before operation there was much abdominal pain, and a history of eating needles, but no physical signs were found at that time. Six weeks before operation patient complained of much pain over the stomach, there was vomiting at times, she was losing weight rapidly, but no tumour could be made out in the abdomen. Four days before operation the pain in the abdomen became general and more severe, and a hard tumour the size of a small orange was made out in the position of the pylorus; the emaciation was by this time marked; the woman now said she had eaten some hat pins, but statements by lunatics cannot always be relied upon. I saw her on November 30th, and from the symptoms and physical signs I thought I might find a malignant growth of the pylorus. I made a free incision into the abdominal cavity, and came down on a large mass of what looked like cancer involving the pyloric one-third of the stomach; on examining this with my finger, I found half a sewing needle. Having now something definite to guide me, I incised the mass, which was three inches thick and covered the whole of the anterior surface of the pyloric end of the stomach; it was hard and cut like scirrhus, the section, however, showing numerous points of hemorrhage with surrounding rings of various shades of yellow to red,—these were no doubt due to punctures by the hat pins. In one of these zones I could feel a sharp point. I deepened the incision and opened up the stomach, felt the head and extracted the first of the ornaments of the female head-gear. The rule which we follow after having removed a stone from the bladder occurred to me, and I decided to make sure there were no more foreign bodies in the stomach, and to explore the whole of this cavity I had to enlarge my opening, and after some delay I extracted the third and then the fourth offending member, the last being firmly fixed in the coats of the stomach. The heads of the pins prevented nature from expelling the unwelcome guests, but she had tried very hard to shut off the general peritoneal cavity by the deposition of so much fibrous tissue. I closed the stomach by three rows of Lembert sutures, and did not drain or wash out the peritoneal cavity. The operation was a long one, and the patient was much collapsed for forty-eight hours: she however recovered rapidly, the mental condition at once improving; she was up and eating light solid food in three weeks, and was discharged from the asylum three months after operation mentally and bodily cured.

It will be observed that the form of mental trouble in most of the cases was one in which we should not expect to find a rapid improvement, even if improvement at all took place under ordinary circumstances.

I am much obliged to Dr. Law Wade and to Dr. Sproat for the histories of some of the cases, and I feel sure that a good deal of the credit and the pleasing results of the cases under their care is due to the way the after-treatment was carried out.

After the reading of Mr. Bush's cases, Dr. J. H. Sproat said that all the cases made good mental recoveries immediately after the operations. The cases of uterine fibroid and the gastrotomy more especially call for remark, as the prognosis of their mental disease was distinctly unfavourable on account of its duration and the constancy of their delusions. Analogous mental recoveries are seen when the insane suffer from severe attacks of acute bodily disease, such as pneumonia or erysipelas; impending dissolution frequently restores the mental condition, and cases of almost hopeless insanity die sane. Such results as accrued from the above cases should determine operation in doubtful cases, and no surgical condition should be allowed to exist in the insane simply because the person is a lunatic. The idea of suicide by swallowing hat-pins was originated in the above patient by reading the gruesome details of an operation for a swallowed hat-pin in one of our up-to-date dailies.—Dr. H. C. Bristowe said he had had some years of lunacy experience, but knew very little about surgical operations on the insane. The only case that came actually under his care was a tracheotomy for malignant disease of œsophagus and larynx; whether there was any improvement he could not say, as the patient did not live long enough. Mr. Bush's cases are, however, of great interest; but it was doubtful whether the mental recovery of those patients was due to the removal of the exciting cause, but rather to the mental effect that the operation had on the individual—the substitution of a healthy for an unhealthy introspection. It is a well established fact that in lunacy improvement takes place in the mental condition during an acute febrile disorder, but it is doubtful whether these cases can be included in the same category.—Dr. J. O. Symes said that the sample of pus submitted by Mr. Bush, in addition to containing the bacillus of blue pus, contained also torulæ and various motile bacteria which were suggestive of its origin from the intestinal tract.

